# When Should We Biopsy? A Risk Factor-Based Predictive Model for EIN and Endometrial Cancer

**DOI:** 10.3390/cancers17233809

**Published:** 2025-11-27

**Authors:** Shina Jang, Sung Ook Hwang

**Affiliations:** Department of Obstetrics and Gynecology, Inha University Hospital, Inha University College of Medicine, Incheon 22212, Republic of Korea

**Keywords:** endometrial cancer, endometrial intraepithelial neoplasia, risk model, predicted probability, obesity, abnormal uterine bleeding, hysteroscopy

## Abstract

Background: The incidence of endometrial cancer (EC) is rising globally across all age groups. Endometrial intraepithelial neoplasia (EIN) is a premalignant lesion that may progress to EC if untreated. A clinical model is needed to efficiently identify women requiring prompt evaluation while avoiding unnecessary invasive procedures. Obesity is a major risk factor, but whether Asian women require a lower body mass index (BMI) cutoff than the World Health Organization (WHO) definition remains debated. This study aimed to develop a multivariable risk prediction model to guide biopsy decisions and determine an appropriate BMI cutoff for predicting EIN/EC risk among Asian women. Methods: This study retrospectively reviewed 1192 women aged ≥18 years who underwent hysteroscopy between 2010 and 2023 at a tertiary hospital. Candidate predictors included patient age, parity, BMI, postmenopausal status, symptom of abnormal uterine bleeding (AUB), diabetes mellitus, hypertension, polycystic ovary syndrome (PCOS), use of oral contraceptives, intrauterine devices, or menopausal hormone therapy, tamoxifen treatment, presence of multiple polyps, and endometrial thickness (EMT) measured by transvaginal ultrasonography. Multivariable logistic regression with stepwise selection identified independent predictors, and model stability and calibration were assessed using 1000 bootstrap resamples. Results: EIN/EC was diagnosed in 55 patients (4.6%). Six independent predictors were identified: postmenopausal status (adjusted odds ratio [aOR] 5.93, 95% CI 2.92–12.04), AUB (aOR 4.07, 1.51–10.97), multiple polyps (aOR 2.49, 1.33–4.66), PCOS (aOR 2.37, 1.08–5.22), BMI (aOR 1.13 per kg/m^2^; 1.84 per +5 kg/m^2^), and EMT (aOR 1.07 per mm, 1.02–1.11). When using categorical cutoffs, Obese II (BMI ≥ 30 kg/m^2^) and markedly increased EMT (≥20 mm) remained significant. Predicted probabilities ranged from 0.3% with no risk factors to 90.9% with all six risk factors present. The final model demonstrated good discrimination (AUC 0.79, 95% CI 0.73–0.86) and excellent calibration on bootstrap validation (mean absolute error 0.005). Conclusions: This six-factor clinical model stratifies individual EIN/EC risk using readily available variables and may guide timely, risk-based biopsy decisions by identifying high-risk patients while minimizing unnecessary procedures in low-risk cases. BMI ≥ 30 kg/m^2^ (WHO obesity threshold) was confirmed as a meaningful cutoff, but external validation is warranted to confirm its generalizability and clinical applicability.

## Simple Summary

Endometrial cancer is becoming increasingly common worldwide, including among younger women. Because abnormal uterine bleeding often has benign causes, timely diagnosis of precancerous or malignant lesions can be challenging. This study analyzed over one thousand women who underwent hysteroscopy to identify clinical features predictive of endometrial intraepithelial neoplasia or cancer. Six independent risk factors were found: postmenopausal, obesity, abnormal uterine bleeding, polycystic ovary syndrome, multiple polyps, and increased endometrial thickness. The developed six-factor model showed good predictive performance and may help clinicians make timely, risk-based decisions for endometrial sampling. A body mass index of 30 kg/m^2^ or higher was confirmed as a meaningful threshold for Asian women, consistent with the World Health Organization definition of obesity.

## 1. Introduction

The global burden of endometrial cancer (EC) has been rising over the past decade, with considerable geographic variation in incidence and mortality rate [1,2]. Notably, population-based data indicate increasing EC incidence among younger women, underscoring the need for risk-based evaluation even when presenting symptoms are nonspecific [3,4]. Endometrial intraepithelial neoplasia (EIN), also referred to as atypical hyperplasia (AH), is recognized as a precursor lesion for EC. EIN is reported to co-occur with EC in approximately one-third of cases and carries an estimated 30% risk of progression to EC if left untreated [5,6,7].

Obesity is recognized as a major preventable determinant in the development of EC. In Asian populations, lower body mass index (BMI) thresholds than the standard World Health Organization (WHO) definition are often used to define metabolic risk, but it remains unclear whether a lower BMI cutoff should also be applied to define elevated EIN/EC risk. Several Asian studies have suggested that the BMI threshold associated with increased EC risk varies across populations [8,9,10]. Only a limited number of investigations have directly examined EIN or identified a consistent BMI threshold that remains independently predictive after adjusting for other factors. Defining a clinically applicable, population-appropriate BMI cutoff for EIN/EC risk therefore remains an open question [5,8,9].

Postmenopausal bleeding (PMB) is widely recognized as a key warning sign of underlying endometrial disease and should be investigated without delay. Beyond PMB, several patient level features are cited as risk factors for EC, including nulliparity, polycystic ovary syndrome (PCOS), and increased endometrial thickness (EMT) on transvaginal ultrasonography [11,12,13,14]. However, the magnitude of risk associated with these factors and optimal clinical thresholds vary widely across studies and populations, including those proposed for EMT. Abnormal uterine bleeding (AUB) in premenopausal and perimenopausal patients is frequently due to benign causes, which may lead to diagnostic delays if risk is underestimated in this younger group.

Accordingly, this study developed a multivariable risk prediction model to generate scenario-based probabilities of EIN/EC and to support decisions regarding the need for endometrial biopsy. The study also determined a clinically applicable, population-appropriate BMI cutoff for discriminating EIN/EC risk in an Asian cohort by analyzing BMI as both a continuous and categorical variable.

## 2. Materials and Methods

This investigation was designed as a retrospective observational review at Inha University Hospital (Incheon, Republic of Korea). Consecutive women aged 18 years or older who underwent hysteroscopic endometrial sampling between 2010 and 2023 were included. Institutional Review Board approval was obtained (IUH-IRB 2025-09-023).

Data on demographic and clinical characteristics were collected through review of electronic medical records. These included patient age; reproductive history (parity); BMI; postmenopausal status; AUB; comorbid conditions such as diabetes mellitus and hypertension; diagnosis of PCOS; ongoing use of oral contraceptives (COCs), intrauterine devices (IUDs), or menopausal hormone therapy (MHT); tamoxifen treatment in women with breast cancer; presence of multiple polyps; and EMT measured by ultrasonography. BMI was calculated from height and weight measured at outpatient visits. The categorization of BMI followed the Asia-Pacific guidelines jointly introduced by the WHO Western Pacific Regional Office (WPRO), the International Obesity Task Force, and the International Association for the Study of Obesity, while also considering recommendations from the WHO Expert Consultation addressing Asian BMI cutoff values [15,16]. Menopause was determined as the absence of menstruation for at least 12 consecutive months in women aged 50 years or older, and in those younger than 50, it was assessed by clinical symptoms and laboratory evidence. AUB and PCOS were identified based on patients’ initial medical records, diagnostic information, self-reported questionnaires, and, for PCOS, ultrasonographic findings. AUB was defined according to the FIGO Menstrual Disorders Group (2009), as non-pregnancy-related uterine bleeding that deviates from normal cyclic patterns in frequency, regularity, duration, or volume, including heavy menstrual bleeding, intermenstrual bleeding or spotting, and irregular cycles [17]. PCOS was defined according to the revised 2003 Rotterdam criteria, requiring at least two of the following: oligo/anovulation, clinical or biochemical hyperandrogenism, or polycystic ovarian morphology [18]. Active tamoxifen therapy was noted if the patient was receiving tamoxifen at the time of evaluation. The presence of two or more polyps visualized during hysteroscopic evaluation, confirmed through operative notes or surgical images, was considered multiple endometrial polyps. EMT was measured via transvaginal (or, when necessary, transrectal) ultrasonography in the mid-sagittal plane as the maximum double-layer thickness (mm). Focal intracavitary lesions (e.g., endometrial polyps, submucosal leiomyomas) were recorded by number and location [19]. Ultrasonographic examinations were performed either by attending physicians or by trained sonographers under physician supervision. All of the above clinical assessments and ultrasonographic examinations were conducted according to standardized protocols.

Indications for hysteroscopic endometrial biopsy included PMB; AUB refractory to medical therapy for at least 1–6 months in premenopausal women; abnormal ultrasound findings such as a suspected endometrial polyp, submucosal myoma, cystic change, or thickened endometrium; and tamoxifen use accompanied by AUB or abnormal endometrial findings on ultrasonography. Exclusion criteria were pregnancy-related bleeding, intraoperative biopsies performed during surgery for ectopic pregnancy or retained placenta, inadequate specimens or absence of endometrial tissue, and follow-up biopsies in patients previously diagnosed with EIN or EC undergoing conservative management. After applying these criteria, 1192 patients remained eligible for analysis.

Hysteroscopic procedures were performed under general anesthesia. Preoperatively, oral misoprostol was administered for cervical softening. Cervical dilation was achieved using Hegar dilators, and the endometrial cavity was evaluated under direct visualization. Most procedures were performed using a gravity-flow system with an infusion height maintained at 1–1.5 m. The average operating time ranged from 10 to 30 min. Depending on the intrauterine findings, therapeutic or diagnostic steps—including removal of polyps, excision of submucosal myomas, or targeted sampling of the endometrium—were undertaken using either a 5 mm rigid hysteroscope (scissors and forceps) or a 10 mm resectoscope fitted with a monopolar loop electrode (Karl Storz, Tuttlingen, Germany).

All endometrial specimens obtained during hysteroscopy were evaluated by board-certified gynecologic pathologists at the institutional pathology department. Histopathologic diagnoses were made according to the WHO classification. Biopsy specimens insufficient for diagnosis (*n* = 7) were excluded from the analysis. The main study endpoint was the identification of clinically relevant endometrial pathology, determined by histological confirmation of EIN/EC. Cases with benign endometrial histology served as the reference group.

**Sample Size Adequacy:** A post hoc assessment of sample size adequacy was conducted for the multivariable model following the framework of Riley et al. for prediction models [20,21]. Assuming a Cox–Snell R^2^ of 0.10, the pmsampsize calculation in R indicated a minimum required sample of 510 patients with 24 outcome events, corresponding to approximately 3.9 events per predictor (for six predictors). This analyzed cohort (N = 1192 with 55 events) exceeded these requirements, yielding about 9.2 events per predictor. This satisfied the recommended shrinkage criterion (≥0.90), and the difference between the model’s apparent and optimism-adjusted R^2^ was ≤0.05, indicating a low risk of overfitting.

**Statistical Analysis:** Baseline characteristics were compared between benign and pathology (EIN/EC) groups using the Wilcoxon rank-sum test (Mann–Whitney U) for continuous variables, chi-square test or Fisher’s exact test for categorical variables, and the Cochran–Armitage trend test for ordinal variables. Any variable demonstrating a univariable association with a *p*-value < 0.10 was included in a multivariable logistic regression model. Adjusted odds ratios (aORs) with 95% CIs were calculated to describe independent predictors. Scenario-based predicted probabilities of EIN/EC were then estimated from the final model to illustrate absolute risk under different clinical scenarios. Internal validation of the final model was performed using 1000 bootstrap resamples to assess model stability and calibration. All statistical procedures were implemented in R (version 4.5.0), with significance defined at *p* < 0.05 (two-sided).

## 3. Results

### 3.1. Cohort and Baseline Characteristics

Among 1192 women who underwent hysteroscopic endometrial sampling, 55 (4.6%) comprised the pathology group (EIN/EC) and 1137 (95.4%) the benign group (Table 1). Compared with the benign group, the pathology group had a higher BMI (median 28.8 vs. 23.2 kg/m^2^, *p* < 0.001) and a greater prevalence of Obese II (BMI ≥ 30 kg/m^2^; 43.6% vs. 9.8%, *p* < 0.001). The pathology group was also more likely to be postmenopausal (32.7% vs. 16.4%, *p* = 0.003), to present with AUB (90.9% vs. 72.0%, *p* = 0.003), and to have PCOS (25.5% vs. 9.6%, *p* < 0.001). Metabolic comorbidities were more frequent (diabetes 14.5% vs. 4.3%, *p* = 0.003; hypertension 29.1% vs. 11.7%, *p* < 0.001). Multiple endometrial polyps were more common (43.6% vs. 22.9%, *p* = 0.001). The median EMT was greater in the pathology group (13.0 mm vs. 10.4 mm, *p* = 0.002), and a markedly thick endometrium (EMT ≥ 20 mm) was also more frequent (23.6% vs. 6.6%, *p* < 0.001).

### 3.2. Histopathology

The histopathological diagnoses for all 1192 patients are summarized in Table 2. Benign endometrial findings accounted for 95.4% of cases (1137 out of 1192), whereas endometrial pathology (EIN/EC) was identified in 4.6% (55/1192). The most common benign diagnosis was endometrial polyp, which was present in 682 cases (57.2% of the total cohort). Other benign findings included disordered proliferative or secretory endometrium (8.1%), submucosal leiomyoma (7.0%), proliferative (7.0%), secretory (6.4%), atrophy (2.3%), endometrial hyperplasia without atypia (1.8%), and a variety of less frequent benign lesions.

Among the 55 cases with premalignant or malignant pathology, AH/EIN was diagnosed in 24 patients (2.0% of the total cohort). Endometrioid endometrial carcinoma was found in 27 patients (grades 1, 2, and 3 in 15, 7, and 5 cases, respectively, totaling 2.3%), and less common histologic types included serous carcinoma (3 cases, 0.2%) and clear cell carcinoma (1 case, 0.1%).

### 3.3. Multivariable Modeling

**Figure 1:**
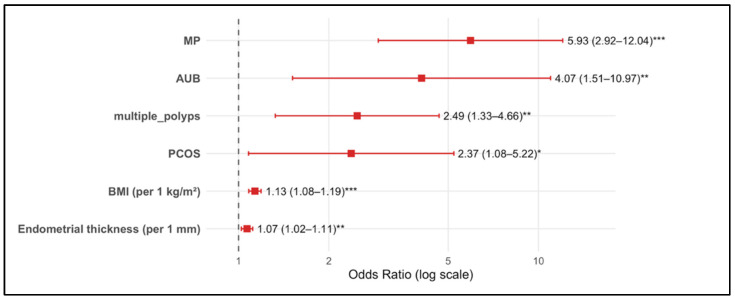
Adjusted odds ratios (aORs) with 95% confidence intervals (CIs) for predictors of EIN/EC from the final multivariable logistic regression model, with BMI (per 1 kg/m^2^) and endometrial thickness (EMT; per 1 mm) entered as continuous predictors. Significance codes: *** *p* < 0.001; ** *p* < 0.01; * *p* < 0.05 (two-sided Wald tests). Abbreviations: MP, postmenopausal; AUB, abnormal uterine bleeding; PCOS, polycystic ovary syndrome; BMI, body mass index.

In the multivariable logistic regression model treating BMI and EMT as continuous variables, six predictors remained independently associated with the likelihood of EIN/EC (Figure 1, Table 3). These independent risk factors were: postmenopausal status (aOR 5.93, 95% CI 2.92–12.04), AUB (aOR 4.07, 95% CI 1.51–10.97), multiple endometrial polyps (aOR 2.49, 95% CI 1.33–4.66), PCOS (aOR 2.37, 95% CI 1.08–5.22), BMI (aOR 1.13 per 1 kg/m^2^ increase, 95% CI 1.08–1.19; aOR 1.84 per +5 kg/m^2^), and EMT (aOR 1.07 per 1 mm increase, 95% CI 1.02–1.11).

**Figure 2:**
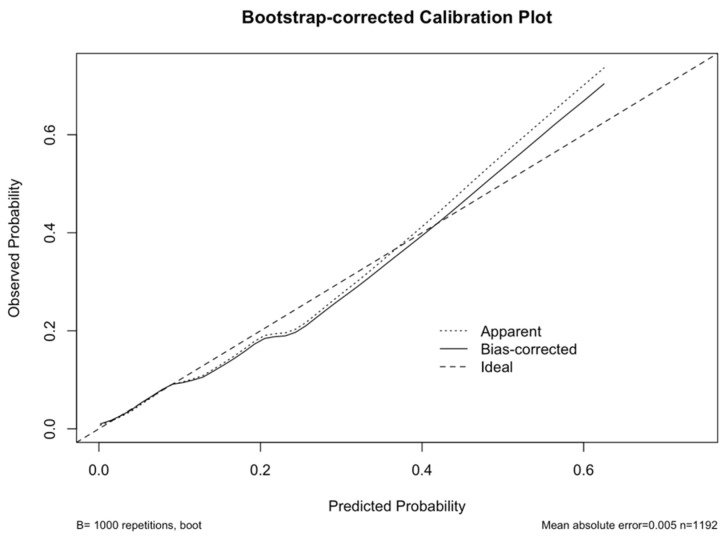
Bootstrap-corrected calibration plot for the final logistic regression model predicting endometrial intraepithelial neoplasia or endometrial cancer (EIN/EC). The dashed line represents perfect calibration (ideal line), the dotted line shows the apparent performance of the model, and the solid line indicates the bias-corrected performance obtained through 1000 bootstrap resamples. The close alignment between the bias-corrected and ideal lines demonstrates excellent agreement between predicted and observed probabilities.

In addition, an alternative model specification was evaluated in which BMI was modeled using WHO Asia-Pacific categories and EMT was analyzed as categorical variables. The categorical model produced results consistent with the continuous-variable analysis (Table 3). Only the Obese II category (≥30 kg/m^2^) showed a significant association with EIN/EC (aOR 5.17, 95% CI 2.43–11.01), whereas Obese I, Overweight, and Underweight were not significant. For EMT, values ≥20 mm were associated with higher risk compared to <15 mm (aOR 2.74, 95% CI 1.23–6.06), while 15–19.9 mm was not different from the reference. In the final analysis, the multivariable logistic regression model demonstrated good discrimination, with an AUC of 0.79 (95% CI 0.73–0.86). The Hosmer–Lemeshow test confirmed adequate model fit (χ^2^ = 2.25, df = 4, *p* = 0.69). Internal validation with 1000 bootstrap resamples showed excellent calibration and model stability, yielding a mean absolute error of 0.005 and a mean squared error of 0.00008, indicating a minimal risk of overfitting (Figure 2).

### 3.4. Predicted Probabilities

**Figure 3:**
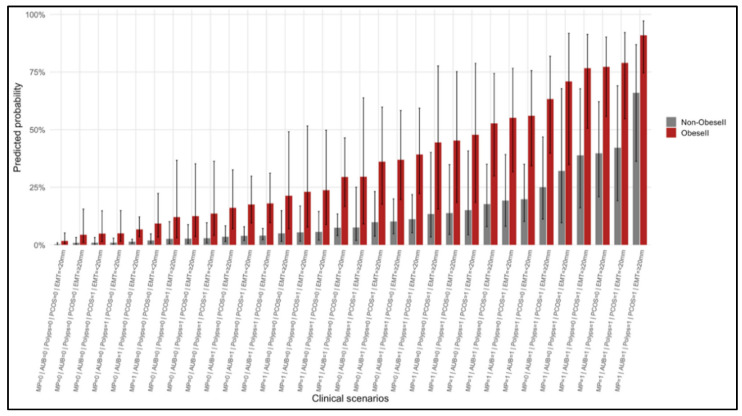
Predicted probability of EIN/EC across clinical scenarios. Bars show model-predicted risk with error bars representing 95% CIs for selected patient profiles, ordered by increasing risk. Gray bars indicate scenarios without Obese II; red bars indicate scenarios with Obese II (BMI ≥ 30 kg/m^2^). Abbreviations: MP, postmenopausal; AUB, abnormal uterine bleeding; PCOS, polycystic ovary syndrome; EMT, endometrial thickness.

Scenario-based predicted probabilities for EIN/EC were generated from the final multivariable model (Figure 3). These estimates demonstrated a wide separation in absolute risk across different clinical profiles. For example, the baseline scenario of a premenopausal woman with no AUB, no PCOS, no multiple polyps, BMI < 30 kg/m^2^, and EMT < 20 mm had an estimated risk of approximately 0.3%. In contrast, a premenopausal woman with AUB (but BMI < 30 and EMT < 20 mm) had an estimated 1.4–1.5% risk. For a postmenopausal woman with AUB, BMI < 30, and EMT < 20 mm, the risk was around 7.5% (95% CI approximately 4.0–13.4%), which increased to 17.7% if the same patient had an EMT ≥ 20 mm. In a premenopausal obese (Obese II) woman with AUB and EMT < 20 mm (and no PCOS or multiple polyps), the predicted risk was about 6.7%, rising to 16.1% if EMT was ≥20 mm. Finally, a high-risk constellation—defined by postmenopausal status, AUB, PCOS, multiple polyps, BMI ≥ 30 kg/m^2^, and EMT ≥ 20 mm—corresponded to an approximate 90.9% predicted probability of EIN/EC.

Overall, the largest increases in risk were associated with postmenopausal status, the presence of AUB, obesity (BMI ≥ 30 kg/m^2^), EMT ≥ 20 mm, and the presence of multiple polyps or PCOS. These trends were consistent with the dose–response relationships observed when BMI and EMT were treated as continuous variables in the model. A complete table of scenario-based predicted probabilities is provided in the Supplementary Materials (Table S1) for reference.

## 4. Discussion

In this study, postmenopausal status, AUB, PCOS, multiple endometrial polyps, higher BMI, and increased EMT were identified as independent predictors of EIN/EC in women undergoing hysteroscopic evaluation. Both BMI and EMT showed clear dose–response associations with risk when analyzed as continuous variables or stratified into categories, with risk particularly elevated at BMI ≥ 30 kg/m^2^ and EMT ≥ 20 mm. These findings reinforce that obesity, endometrial thickening, and other clinical factors contribute significantly to the risk of premalignant or malignant endometrial pathology, and they should be considered when assessing patients for possible endometrial sampling. 

**Obesity and EC risk.** Obesity and the risk of EC have been extensively studied. Large-scale cohorts and meta-analyses consistently indicate that every 5 kg/m^2^ increment in BMI confers a 1.5–1.8-fold elevation in risk [22,23]. Furthermore, The World Cancer Research Fund has identified body fatness as a definitive causal factor for EC [24]. In the present analysis, each 1 kg/m^2^ rise in BMI was linked with an aOR of 1.13 (95% CI 1.08–1.19), which corresponds to an approximately 1.8-fold higher odds of EIN/EC per 5 kg/m^2^. This is consistent with prior evidence and confirms that adiposity contributes independently to endometrial carcinogenic risk.

**Endometrial thickness.** EMT likewise showed a dose–response relationship with pathology risk. In this study, each 1 mm increase in EMT was associated with an aOR of 1.07 (95% CI 1.02–1.11) for EIN/EC. When categorized, an EMT ≥ 20 mm conferred a higher risk of EIN/EC (approximately 2.7-fold compared to <15 mm), whereas intermediate thickness (15–19.9 mm) did not significantly differ from the <15 mm reference. In the context of PMB, an EMT ≤ 4 mm is widely accepted as a safe threshold below which significant pathology is unlikely [25,26]. In premenopausal or perimenopausal women, however, proposed EMT cutoffs (often between 8–14 mm) have shown inconsistent performance for cancer prediction [27]. The present findings indicate that markedly increased EMT (≥20 mm) can serve as a red flag for endometrial neoplasia in a referred cohort, whereas moderate thickening appears to confer little additional risk once other variables are taken into account.

**Other factors.** Within the model, AUB and postmenopausal status proved to be the most influential factors associated with EIN/EC risk. This aligns with clinical guidance that any bleeding in a postmenopausal woman warrants prompt evaluation and with data showing higher pretest probabilities of malignancy in symptomatic populations [25]. Notably, in this cohort AUB remained significantly associated with EIN/EC despite adjustment for BMI, EMT, and polyp status. This indicates that abnormal bleeding is best regarded as a direct clinical manifestation of underlying pathology rather than an indirect reflection of other factors.

**PCOS** remained independently associated with EIN/EC, in line with recent meta-analyses reporting a higher risk of EC among women with PCOS [11,28]. The underlying mechanisms are thought to involve chronic anovulation with prolonged unopposed estrogen exposure, insulin resistance with compensatory hyperinsulinemia, low-grade inflammation, and features of progesterone resistance [28,29]. These results highlight the need to consider PCOS in risk-stratified diagnostic approaches for women presenting with AUB. 

**Multiple endometrial polyps** were also an independent predictor of EIN/EC. Although most polyps are benign, previous studies have reported that the presence of multiple polyps is associated with a higher risk of atypical hyperplasia or carcinoma compared with cases involving a single polyp [30,31]. This association may reflect an increased proliferative potential associated with greater lesion volume or an inflammatory background that promotes endometrial neoplastic transformation [31]. 

Several variables showed associations in univariable analysis but did not remain significant predictors in the multivariable model. Nulliparity, for example, lost significance after adjustment. This finding may be explained by overlapping mechanisms with PCOS and AUB, such as anovulatory cycles and prolonged estrogen exposure, together with the limited number of events, which reduced the ability to detect small independent effects. Similarly, diabetes mellitus and hypertension were associated with EIN/EC in unadjusted comparisons but were no longer significant after controlling for BMI, consistent with literature suggesting that the apparent links between these metabolic conditions and EC risk are largely confounded or mediated by adiposity and related metabolic disturbances [32,33].

Recent studies have developed predictive models for endometrial malignancy using various approaches. The BLUSH model combined clinical, ultrasonographic, and hysteroscopic features with high diagnostic accuracy, while the SIR-En model integrated systemic inflammatory indices with EMT and showed moderate performance [34,35]. This study adds to this growing body of work by presenting a model based on routinely available clinical factors, which demonstrated good discrimination and satisfactory calibration after internal validation.

**Strengths and limitations.** A key strength of this study is the relatively large sample size for a hysteroscopy-based analysis, together with histopathological confirmation of all outcomes. Risk factors were assessed using both continuous and categorical specifications, and BMI categories were defined according to region-specific criteria, which allowed identification of a potential threshold relevant to risk. The multivariable model was carefully evaluated to minimize overfitting, and it demonstrated favorable performance (AUC = 0.79; Hosmer–Lemeshow χ^2^ = 2.25, *p* = 0.69). Furthermore, internal bootstrap validation confirmed excellent calibration and model stability, supporting the reliability of these findings despite the absence of external validation.

Several limitations should be noted. The study was retrospective and performed at a single tertiary referral hospital, potentially limiting generalizability and introducing selection bias, since only women referred for hysteroscopy were included. The number of EIN/EC events was modest (*n* = 55). Although this exceeded the calculated requirement from the sample size analysis, it nonetheless constrained model complexity and reduced the precision of some estimates. External validation was not available, leaving uncertainty about the model’s performance in other populations. In addition, EMT in premenopausal women was measured without accounting for cycle phase, which may have introduced variability. These limitations indicate that caution is warranted when interpreting the absolute risk estimates, and they highlight the importance of future studies to validate and extend these findings.

## 5. Conclusions

In this cohort, BMI ≥ 30 kg/m^2^ and EMT ≥ 20 mm, along with postmenopausal status, AUB, PCOS, and multiple endometrial polyps, were independently associated with EIN/EC. A predictive model incorporating these six factors produced estimated probabilities ranging from <1% to nearly 91%, demonstrating wide variation in absolute risk clinical profiles. These findings suggest that common clinical variables can be integrated for individualized risk estimation and may aid in the risk stratification of women undergoing hysteroscopic evaluation. External validation of this model in diverse populations or clinical settings is warranted to confirm its generalizability and applicability.

## Figures and Tables

**Table 1:** Baseline demographic and clinical characteristics by outcome.

Group	Benign (N = 1137)	EIN/EC (N = 55)	Total (N = 1192)	*p*
Age	43.0 [36.0;49.0]	44.0 [34.5;61.0]	43.0 [36.0;49.0]	0.377
Postmenopausal status				0.003
no	951 (83.6%)	37 (67.3%)	988 (82.9%)	
yes	186 (16.4%)	18 (32.7%)	204 (17.1%)	
BMI (kg/m^2^)	23.2 [21.1;26.2]	28.8 [22.8;33.0]	23.3 [21.2;26.6]	<0.001
BMI_WPRO ^1^				<0.001
Under weight	53 (4.7%)	2 (3.6%)	55 (4.6%)	
Normal	491 (43.2%)	13 (23.6%)	504 (42.3%)	
Overweight	200 (17.6%)	4 (7.3%)	204 (17.1%)	
Obese I	282 (24.8%)	12 (21.8%)	294 (24.7%)	
Obese II	111 (9.8%)	24 (43.6%)	135 (11.3%)	
Nulliparous				0.015
no	824 (72.5%)	31 (56.4%)	855 (71.7%)	
yes	313 (27.5%)	24 (43.6%)	337 (28.3%)	
AUB				0.003
absent	318 (28.0%)	5 (9.1%)	323 (27.1%)	
present	819 (72.0%)	50 (90.9%)	869 (72.9%)	
PCOS				<0.001
no	1028 (90.4%)	41 (74.5%)	1069 (89.7%)	
yes	109 (9.6%)	14 (25.5%)	123 (10.3%)	
Diabetes				0.003
absent	1088 (95.7%)	47 (85.5%)	1135 (95.2%)	
present	49 (4.3%)	8 (14.5%)	57 (4.8%)	
Hypertension				<0.001
absent	1004 (88.3%)	39 (70.9%)	1043 (87.5%)	
present	133 (11.7%)	16 (29.1%)	149 (12.5%)	
COCs/IUD/MHT use				0.213
no	1028 (90.4%)	53 (96.4%)	1081 (90.7%)	
yes	109 (9.6%)	2 (3.6%)	111 (9.3%)	
Multiple polyps				0.001
absent	877 (77.1%)	31 (56.4%)	908 (76.2%)	
present	260 (22.9%)	24 (43.6%)	284 (23.8%)	
Tamoxifen use				1.000
no	1077 (94.7%)	53 (96.4%)	1130 (94.8%)	
yes	60 (5.3%)	2 (3.6%)	62 (5.2%)	
EMT (mm)	10.4 [7.1;14.0]	13.0 [8.4;19.2]	10.5 [7.2;14.2]	0.002
EMT (category)				<0.001
<15 mm	898 (79.0%)	33 (60.0%)	931 (78.1%)	
15 mm–19.9 mm	164 (14.4%)	9 (16.4%)	173 (14.5%)	
≥20 mm	75 (6.6%)	13 (23.6%)	88 (7.4%)	

Abbreviations: BMI, body mass index; AUB, abnormal uterine bleeding; PCOS, polycystic ovary syndrome; EIN, endometrial intraepithelial neoplasia; EC, endometrial carcinoma; EMT, endometrial thickness; COCs, combined oral contraceptives; IUD, intrauterine device (levonorgestrel-releasing intrauterine system, LNG-IUS); MHT, menopausal hormone therapy; WPRO, World Health Organization Western Pacific Regional Office. ^1^ BMI category (WPRO): categorized according to the WHO Western Pacific Regional Office (WPRO) classification: Underweight (<18.5 kg/m^2^), Normal (18.5–22.9 kg/m^2^), Overweight (23.0–24.9 kg/m^2^), Obese I (25.0–29.9 kg/m^2^), and Obese II (≥30 kg/m^2^).

**Table 2:** Histopathological diagnoses in the study population (N = 1192).

Benign group	1137 (95.4%)
Endometrial polyp	682 (57.2%)
Disordered proliferative/secretory phase	96 (8.1%)
Submucosal leiomyoma	84 (7.0%)
Proliferative phase	84 (7.0%)
Secretory phase	76 (6.4%)
Atrophy	27 (2.3%)
Endometrial hyperplasia without atypia (EH)	21 (1.8%)
Inactive	17 (1.4%)
Acute and chronic endometritis	16 (1.3%)
Glandular and stromal breakdown	12 (1.0%)
Adenomyomatous polyp	7 (0.6%)
Others *	15 (1.3%)
Premalignant/malignant group	55 (4.6%)
Atypical hyperplasia/endometrial intraepithelial neoplasia (AH/EIN)	24 (2.0%)
Endometrioid adenocarcinoma grade 1 (EC)	15 (1.3%)
Endometrioid adenocarcinoma grade 2 (EC)	7 (0.6%)
Endometrioid adenocarcinoma grade 3 (EC)	5 (0.4%)
Serous carcinoma	3 (0.2%)
Clear cell carcinoma	1 (0.1%)

Counts (percentages) are shown for the entire cohort. * Benign “Others” include benign endometrial tissue (*n* = 8), decidualized stroma (*n* = 2), progestin effect (*n* = 2), granulomatous inflammation with necrosis (*n* = 2), and epithelioid mesenchymal tumor (*n* = 1).

**Table 3:** Multivariable logistic regression results (final model), comparing continuous vs. categorical specifications of BMI and EMT.

Variable	Continuous Model		Categorical Model	
	aOR (95% CI)	*p*	aOR (95% CI)	*p*
Postmenopausal status	5.93 (2.92–12.04)	<0.001	5.96 (2.87–12.37)	<0.001
BMI (per 1 kg/m^2^)	1.13 (1.08–1.19)	<0.001		
BMI_WPRO: Obese II vs. Normal			5.17 (2.43–11.01)	<0.001
BMI_WPRO: Under vs. Normal			1.50 (0.32–7.07)	0.610
BMI_WPRO: Obese I vs. Normal			1.17 (0.50–2.70)	0.720
BMI_WPRO: Over vs. Normal			0.64 (0.20–2.07)	0.461
AUB	4.07 (1.51–10.97)	0.005	4.11 (1.51–11.2)	0.006
Multiple polyps	2.49 (1.33–4.66)	0.005	2.97 (1.59–5.56)	<0.001
PCOS	2.37 (1.08–5.22)	0.032	2.91 (1.36–6.22)	0.006
EMT (mm)	1.07 (1.02–1.11)	0.004		
EMT ≥ 20 mm vs. EMT < 15 mm			2.74 (1.23–6.06)	0.013
15 mm–19.9 mm vs. EMT < 15 mm			1.11 (0.49–2.49)	0.804

Abbreviations: aOR, adjusted odds ratio; BMI, body mass index; WPRO, World Health Organization Western Pacific Regional Office; AUB, abnormal uterine bleeding; PCOS, polycystic ovary syndrome; EMT, endometrial thickness.

## Data Availability

The data presented in this study are available on request from the corresponding author. The data are not publicly available due to privacy restrictions.

## Supplementary Materials

The following supporting information can be downloaded at: https://www.mdpi.com/article/10.3390/cancers17233809/s1, Table S1: Scenario-based predicted probabilities of EIN/EC according to clinical risk factors.

## Abbreviations

The following abbreviations are used in this manuscript:

AH	Atypical Hyperplasia
AUB	Abnormal Uterine Bleeding
BMI	Body Mass Index
EC	Endometrial Cancer
EIN	Endometrial Intraepithelial Neoplasia
EMT	Endometrial Thickness
MHT	Menopausal Hormone Therapy
PCOS	Polycystic Ovary Syndrome
PMB	Postmenopausal Bleeding
WPRO	World Health Organization Western Pacific Regional Office
