# Supplementary material for: When Should We Biopsy? A Risk Factor-Based Predictive Model for EIN and Endometrial Cancer
Source: Cancers (Basel). 2025 Nov 27;17(23):3809. doi: 10.3390/cancers17233809 (PMC12691479; doi:10.3390/cancers17233809)
Supplement: Supplementary file 1 [file cancers-17-03809-s001.zip › cancers-3928774-supplementary.pdf]

Supplementary Table S1.

Scenario-based predicted probabilities of EIN/EC according to clinical risk factors

no.	Obese II (BMI≧30)	Menopause	AUB	multiple polyps	PCOS	EMT≧20 mm	Predicted	probability (95% CI)
1	0	0	0	0	0	0	0.30%	(0.1–1.0)
2	0	0	0	0	0	1	0.90%	(0.2–3.3)
3	0	0	0	0	1	0	1.00%	(0.3–3.2)
4	0	0	0	1	0	0	1.00%	(0.3–3.0)
5	0	0	1	0	0	0	1.40%	(0.8–2.4)
6	1	0	0	0	0	0	1.70%	(0.6–5.2)
7	0	1	0	0	0	0	1.90%	(0.8–4.8)
8	0	0	0	0	1	1	2.60%	(0.6–10.1)
9	0	0	0	1	0	1	2.70%	(0.8–8.8)
10	0	0	0	1	1	0	3.00%	(0.9–9.6)
11	0	0	1	0	0	1	3.60%	(1.5–8.3)
12	0	0	1	0	1	0	3.90%	(1.9–7.9)
13	0	0	1	1	0	0	4.10%	(2.3–7.2)
14	1	0	0	0	0	1	4.50%	(1.2–15.5)
15	1	0	0	0	1	0	4.90%	(1.5–14.8)
16	0	1	0	0	0	1	5.00%	(1.5–14.9)
17	1	0	0	1	0	0	5.10%	(1.6–14.9)
18	0	1	0	0	1	0	5.50%	(1.6–16.9)
19	0	1	0	1	0	0	5.70%	(2.1–14.6)
20	1	0	1	0	0	0	6.70%	(3.6–12.2)
21	0	1	1	0	0	0	7.50%	(4.0–13.4)
22	0	0	0	1	1	1	7.50%	(1.9–25.0)
23	1	1	0	0	0	0	9.20%	(3.5–22.4)
24	0	0	1	0	1	1	9.80%	(3.8–23.2)
25	0	0	1	1	0	1	10.20%	(4.9–20.0)
26	0	0	1	1	1	0	11.10%	(5.3–21.9)
27	1	0	0	0	1	1	12.10%	(3.2–36.7)
28	1	0	0	1	0	1	12.50%	(3.6–35.2)
29	0	1	0	0	1	1	13.40%	(3.4–40.2)
30	1	0	0	1	1	0	13.60%	(4.2–36.3)
31	0	1	0	1	0	1	13.80%	(4.6–34.8)
32	0	1	0	1	1	0	15.10%	(4.4–40.8)
33	1	0	1	0	0	1	16.10%	(7.1–32.6)

34	1	0	1	0	1	0	17.50%	(9.5–29.9)
35	0	1	1	0	0	1	17.70%	(7.9–35.0)
36	1	0	1	1	0	0	18.00%	(9.6–31.1)
37	0	1	1	0	1	0	19.20%	(8.1–39.2)
38	0	1	1	1	0	0	19.80%	(10.2–35.0)
39	1	1	0	0	0	1	21.30%	(7.1–49.1)
40	1	1	0	0	1	0	23.10%	(7.8–51.6)
41	1	1	0	1	0	0	23.70%	(8.9–49.8)
42	0	0	1	1	1	1	25.00%	(11.2–46.8)
43	1	1	1	0	0	0	29.40%	(16.7–46.4)
44	1	0	0	1	1	1	29.60%	(9.1–63.8)
45	0	1	0	1	1	1	32.10%	(9.6–67.8)
46	1	0	1	0	1	1	36.10%	(17.6–59.8)
47	1	0	1	1	0	1	36.90%	(19.7–58.3)
48	0	1	1	0	1	1	38.80%	(16.1–67.8)
49	1	0	1	1	1	0	39.30%	(22.3–59.3)
50	0	1	1	1	0	1	39.70%	(20.9–62.1)
51	0	1	1	1	1	0	42.10%	(19.2–69.1)
52	1	1	0	0	1	1	44.40%	(15.5–77.6)
53	1	1	0	1	0	1	45.30%	(18.5–75.2)
54	1	1	0	1	1	0	47.80%	(18.4–78.8)
55	1	1	1	0	0	1	52.70%	(29.9–74.3)
56	1	1	1	0	1	0	55.20%	(31.7–76.6)
57	1	1	1	1	0	0	56.00%	(34.4–75.6)
58	1	0	1	1	1	1	63.30%	(39.8–81.8)
59	0	1	1	1	1	1	66.00%	(36.3–86.9)
60	1	1	0	1	1	1	71.00%	(34.8–91.8)
61	1	1	1	0	1	1	76.60%	(50.6–91.3)
62	1	1	1	1	0	1	77.30%	(55.8–90.2)
63	1	1	1	1	1	0	79.00%	(54.7–92.1)
64	1	1	1	1	1	1	90.90%	(74.6–97.2)

Abbreviations: BMI, body mass index; AUB, abnormal uterine bleeding; PCOS, polycystic ovary syndrome; EMT, endometrial thickness.
